# Occupational social class trajectories in physical functioning among employed women from midlife to retirement

**DOI:** 10.1186/s12889-019-7880-0

**Published:** 2019-11-14

**Authors:** Eero Lahelma, Olli Pietiläinen, Tarani Chandola, Martin Hyde, Ossi Rahkonen, Tea Lallukka

**Affiliations:** 1 Department of Public Health, University of Helsinki, P.O. Box 20, (Tukholmankatu 8 2B), 00014 Helsinki, Finland; 2 Cathie Marsh Institute and Social Statistics, University of Manchester, Oxford Rd, Manchester, M13 9PL UK; 3 Centre for Innovative Ageing, Swansea University, Singleton Park, Swansea, SA2 8PP UK; 4 Finnish Institute of Occupational Health, Helsinki, Finland

**Keywords:** Physical functioning, Trajectories, Social class, Retirement, Women

## Abstract

**Background:**

Prior analyses of class differences in health trajectories among employees have often omitted women and transitions to retirement. We examined social class trajectories in physical functioning among Finnish female employees from midlife to retirement age, and whether transitions to retirement modified these trajectories.

**Methods:**

Data were derived from mail surveys at Phases 1–3 (2000–2012) among employees of the City of Helsinki, Finland, aged 40–60 at baseline (*n* = 8960, 80% women, response rates 69–83%). We included respondents to any of the Phases 1–3 aged 40–72 (*n* = 6976). We distinguished higher and lower social classes, and employment statuses, i.e. employed, mandatorily retired and disability-retired. Short Form 36 physical component summary was used to measure physical functioning. Mixed-effect growth curve models were used to assess the association of social class and employment status with functioning over age.

**Results:**

For employed women, physical functioning deteriorated faster in the lower than in the higher class, with class trajectories widening in ages 40–65. After mandatory retirement, functioning deteriorated in both classes, whereas after disability retirement, functioning improved. Across employment statuses, functioning converged at older ages, and the disability-retired caught up with the better functioning of the employed and mandatorily retired. Employment status modified the trajectories, as among the continuously employed and mandatorily retired women functioning deteriorated, but among the disability-retired, trajectories improved and reached a similar level with employed and mandatorily retired women. Social class inequalities remained in all employment status groups.

**Conclusions:**

Overall, our results suggest evidence for the cumulative disadvantage model, with accumulating work exposures among lower classes potentially contributing to their trajectories of ill health.

## Background

Affluent societies, including Finland, are undergoing a major demographic transition, as large post-war baby-boomer generations face the end of their work career and transition to retirement [1–3]. This transition coupled with longer life expectancy has led governments to be concerned about extending work careers. Ageing employees’ chances to continue in paid employment are dependent on their health and functioning [4, 5]. Furthermore, those in lower social classes suffer from poorer functioning and run a higher risk of transition to disability retirement than their upper class counterparts [6–8]. Our focus is on changes in occupational social class differences in physical functioning among Finnish female employees from midlife to retirement age, and, in particular, on whether transitions to mandatory or disability retirement modifies the changes as the women age.

Two theoretical models have been proposed to predict socioeconomic inequalities in health trajectories over the life course. The first one, the cumulative disadvantage theory, suggests that socioeconomic inequalities in health tend to widen as disadvantage accumulates in an unequal way [7, 9–12]. The second one, the age-as-leveller theory, suggests that socioeconomic inequalities in health tend to narrow in older age, as the effects of disadvantage weaken due to retirement and advancing biological frailty [10, 11].

Some studies have examined socioeconomic differences in health trajectories in general. A study on the British Whitehall II cohort among non-manual employees reported that physical functioning declined and social class inequalities widened towards early old age following the cumulative disadvantage theory [13]. In a study from the United States, educational inequalities in physical functioning remained until early old age, but narrowed in later old age following the age-as-leveller theory [14]. Self-rated health [15–17] and cardiovascular risks [18] have equally shown widening socioeconomic trajectories. Two studies were stratified by gender and they showed persisting socioeconomic differences among both men and women [17, 18]. A review reported persistent socioeconomic inequalities in self-rated health trajectories, with little evidence for cumulative disadvantage and widening inequalities over adult life [19]. Overall, there is some evidence for the cumulative disadvantage theory and widening health inequalities, but less for the age-as-leveller theory and narrowing health inequalities in older ages.

Some other studies have examined socioeconomic trajectories in health further by considering the routes to exit paid employment. In the Whitehall II cohort, physical functioning deteriorated less in the lower class retired than in the higher class retired in older age groups, but the deterioration and widening inequalities remained [13]. In another Whitehall-based study, functioning was poorer for the ill health-retired than for the statutory-retired, with minor differences between social classes [20]. In a French occupational cohort, self-rated health deteriorated towards transition to retirement but improved after that. The deterioration was stronger in the lower social classes, whereas after transition to retirement there was no additional deterioration for these classes [21]. In these studies including retirement transition, gender was adjusted for and possible differences between men and women could not be judged. A review concluded that statutory retirement showed stronger beneficial health effects in higher than in lower classes [22]. However, the picture was heterogeneous and, due to lacking evidence, the effects of disability retirement could not be included.

A main limitation of the existing studies is that they have included men only, male-dominated data, or adjusted for gender in the analyses. In the Finnish retirement schemes, the proportion of women among mandatory retirees as well as disability retirees is equal to men, i.e. 52% [23]. Women tend to occupy more disadvantaged class positions and the existing studies examining health trajectories largely in male cohorts may underestimate class differences in health.

Retirement is the key route to exit paid employment and previous studies suggest that this route is unlikely uniform [20–22]. Mandatory age-based and disability-based retirement signify divergent exit routes, with potential effects on the socioeconomic inequalities in physical functioning trajectories. There are further limitations in the existing studies. The Whitehall-based studies exclude manual workers. In the French occupational cohort, retirement age with full pension award is low, i.e. 50–60 years, and physical functioning is not measured [24]. Both cohorts are male-dominated, and studies reporting on women are lacking. Trajectories in functioning are shaped by age, social class and employment status, but their joint importance for functioning remains poorly understood. Therefore, three-variable interaction between age, social class and employment status in the study of trajectories in functioning needs to be studied [22].

The route to retirement is a critical transition that likely shapes the social class trajectories in health [25]. We extend, firstly, the prior studies by focusing on women. Secondly, we extend the studies on social class inequalities in functioning over ageing by examining the modifying effect of employment status, i.e. continuous paid employment, mandatory retirement due to age and premature retirement due to disability. We also broaden the cumulative disadvantage and age-as-leveller models, as they predict widening or narrowing socioeconomic inequalities in health trajectories in general, whereas we examine further whether the widening or narrowing in functioning by social class varies between divergent employment statuses.

Our specific aims were:

1. To examine whether social class trajectories in physical functioning widen, narrow or remain stable among female employees from midlife to retirement age and beyond; and
2. To examine whether employment status, i.e. continuing in paid employment, transition to mandatory retirement or retirement due to disability, modifies the social class trajectories in functioning.

## Methods

### Context of the study

Our study is part of the Helsinki Health Study on the staff of the City of Helsinki, Finland, originally employed in 2000–2002. The City of Helsinki has remained economically stable and even during the 2008–2009 international financial crisis redundancies were avoided. The work careers are typically long and the annual turnover is low (ca. 4%). The municipality is in charge of general local administration, health and social welfare, education and culture, public transport, and technical services. Helsinki is the largest single employer in Finland, with a staff around 40,000. All employees share similar personnel policies, administration and registration, as well as access to occupational health care. Three quarters of the staff are women, and the median age is 45 years. Over 80% are permanently employed, and 88% have full-time jobs. There are hundreds of occupations from managers and professionals to lower non-manual employees to manual workers [26, 27].

### Data sources

The Helsinki Health Study baseline mail surveys were collected at Phase 1 in 2000, 2001 and 2002 among employees of City of Helsinki, who turned 40, 45, 50, 55 and 60 years in each year. The target population included 13,344 employees of whom 8960 responded at Phase 1 [26]. In this study, we focus on female employees only (80% of the respondents). Follow-up surveys were mailed to all Phase 1 responders. The response rate among women was 69% at Phase 1 in 2000–2002 (*n* = 7168), 83% at Phase 2 in 2007 (*n* = 5980) and 78% at Phase 3 in 2012 (*n* = 5558). Women with complete information on occupational social class, employment status and functioning at any Phase 1–3 (*n* = 6976) were included (Table 1). We examined ages from 40 to 72 years.

**Table 1:** Distributions of social class and employment status among women

	Phases 1–3			
Social class	%	N		
Upper class	46	3197		
Lower class	54	3779		
All	100	6976		
	Phase 1	Phase 2	Phase 3	Phases 1–3
Employment status	%	%	%	N
Employed	100	80	58	4643
Mandatorily retired	–	16	35	1934
Disability-retired	–	4	7	399
All	100	100	100	6976

### Measures

Our socioeconomic measure was occupation-based social class, class for short, which is suitable for an employee cohort. Information on social class at baseline was derived from the City of Helsinki personnel register for consenters to such data linkage (78%). For the rest, class was obtained from the questionnaire. For non-manual occupations, we followed the City of Helsinki socioeconomic classification and for manual occupations Statistics Finland classification [28]. Those lacking information on social class were omitted (*n* = 172). Following previous studies [15, 16], we collapsed managers, professionals and semi-professionals into higher class (46%), and routine non-manual employees and manual workers into lower class (54%) (Table 1). Among those who remained employed at Phases 1–3, only 3% moved from the higher to the lower class and 8% from the lower to the higher class (Table 2).

**Table 2:** Social class at Phase 1 and Phase 3 among employed women

	Social class at Phase 3				
	Upper class	Lower class	Other	Non-respondents	All
	%	%	%	%	%
Social class at Phase 1					
Upper class	67	3	2	29	100
Lower class	8	51	0	40	100

Retirement type was asked in the questionnaire and all non-retired were employed in this occupational cohort. Our employment status variable was classified into: 1) Employed; 2) Retired due to mandatory age; and 3) Retired due to disability. In the Finnish retirement schemes, employees are entitled to either mandatory earnings-related retirement based on age or retirement based on disability [23, 29, 30]. Until 2005, the age for mandatory retirement was 65 years, but after that, it has been flexible from 63 to 68 years, meaning that the oldest employees are 67 years. Transition to retirement at varying ages showed negligible social class differences. We included all mandatory retirements from age 55 on. Disability retirement can be granted until 63 years after assessment of work ability based on medical diagnosis, and information e.g. on working conditions and age. For full disability retirement, work ability should be reduced by 60% and for partial retirement by 40%. All our participants were employed at phase 1 (Table 1). At Phase 2, 16% were mandatorily retired and 4% disability-retired; at Phase 3, the figures were 35 and 7%. Our data contain 1934 mandatory retirements and 399 disability retirements.

Employment status was used as a time-varying variable in the analyses, and therefore the trajectories of functioning among the mandatorily retired and the disability-retired reflect changes in functioning after the retirement transition, not retroactively before the transition. However, as disability retirements are automatically converted to old-age retirements at age 63, those undergoing such conversion were continuously treated as disability-retired, since the conversion is rather a legal than a substantial change in the employee status. The class difference for being continuously employed was minor, but after ages 55–60 the proportion of mandatorily retired was lower and the proportion of disability-retired was higher for the lower class.

Our health outcome was physical functioning, functioning for short, measured by the Short Form 36 (SF-36) health inventory at each Phase 1–3 [31, 32]. Physical functioning is well suited for our purposes as it indicates health-related limitations in the work and non-work setting. SF-36 comprises 36 items and from these, continuous physical component summary (PCS) scores are calculated. Higher scores indicate better functioning, with a range of 0–100, a mean of 50 and a standard deviation of 10 in the US general population. A difference of 3 scores or more is regarded as clinically significant [33]. SF-36 has high construct validity, overall internal consistency and test-retest reliability [31, 32].

### Statistical analyses

**Fig. 1:**
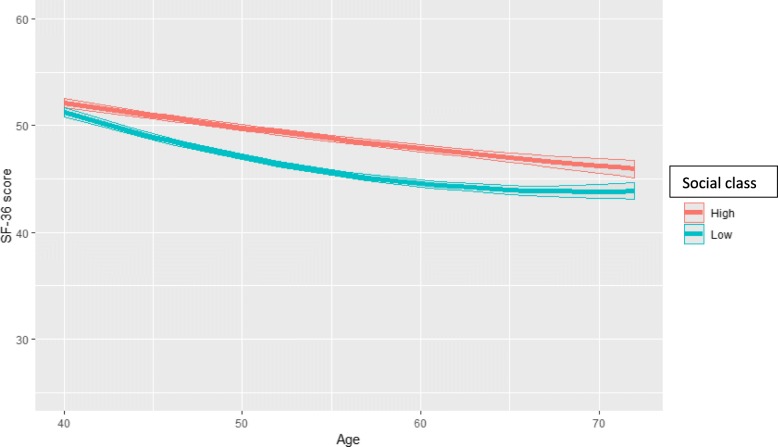
Physical functioning (predicted SF-36 PCS score and 95% confidence intervals) by age and social class among all women aged 40–72 (model = age*social class)

**Fig. 2:**
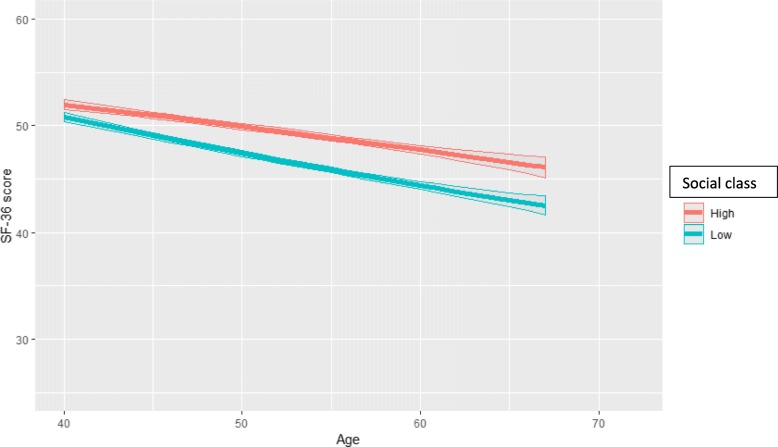
Physical functioning (predicted SF-36 PCS score and 95% confidence intervals) by age and social class among employed women aged 40–72 (model = age*social class*employment status)

**Fig. 3:**
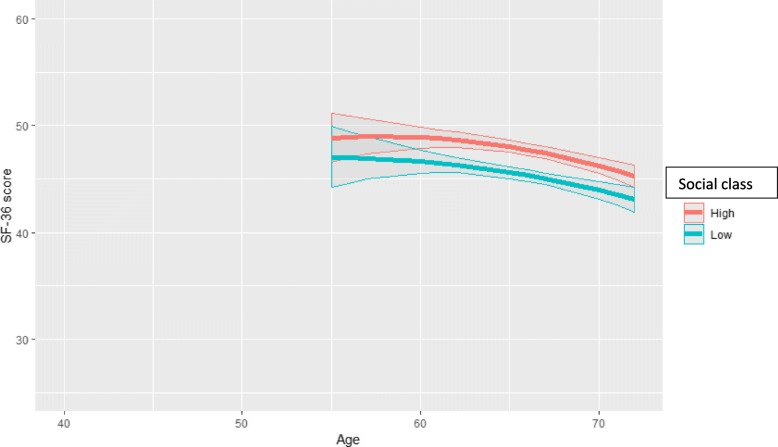
Physical functioning (predicted SF-36 PCS score and 95% confidence intervals) by age and social class among mandatorily retired women aged 55–72 (model = age*social class*employment status)

**Fig. 4:**
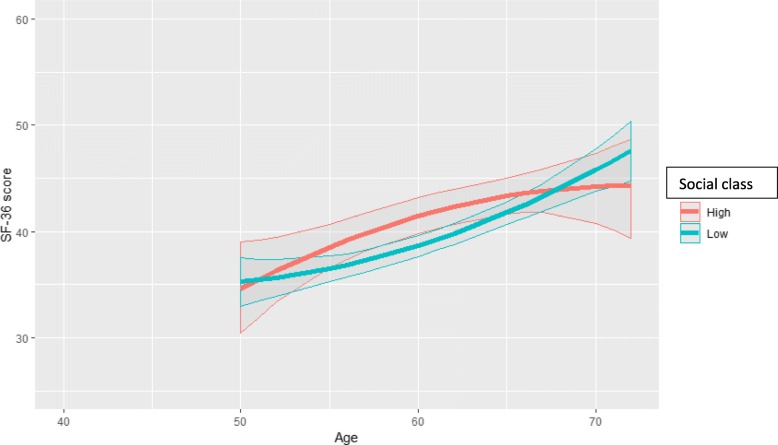
Physical functioning (predicted SF-36 PCS score and 95% confidence intervals) by age and social class among disability-retired women aged 50–72 (model = age*social class*employment status)

Mixed-effect growth curve models were used to assess the association of social class and employment status with SF-36 scores over age. This method enables us to measure the common time trends in functioning while simultaneously taking into account the individual variability around these time trends [34]. Firstly, to examine the overall class trajectories in functioning, we fitted a model with SF-36 PCS score as independent variable, and age, square of age, social class, two-variable interaction between social class and age, and two-variable interaction between social class and square of age as fixed effects. Participant-specific intercept and slope for age, indicating change in SF-36 score, were included as random effects. Secondly, to examine whether employment status, i.e. continuous employment, mandatory retirement or disability retirement, modifies the class trajectories in functioning as the participants become older, we fitted a model including the same variables as in the previous model, with additionally employment status and three-variable interaction between social class, employment status and age as well as three-variable interaction between social class, employment status and square of age included as fixed effects. SF-36 scores were predicted from models at each age for all combinations of social class and employment status. Figure 1 presents overall SF-36 scores by class over age. Figures 2, 3 and 4 present SF-36 scores stratified for each employment status group by class over age. We calculated 95% confidence intervals (CI) for the model predictions using bootstrapping.

## Results

The first analysis fitted a two-variable interaction term between age and social class, showing PCS trajectories by class in the full cohort of all studied women (Fig. 1). The trajectories declined for both classes, with the predicted PCS score deteriorating for the higher class from 52.1 (CI 51.7–52.5) at age 40 to 46.0 (CI 45.1–46.7) at age 72, and for the lower class from 51.2 (50.8–51.7) at age 40 to 43.8 (43.1–44.6) at age 72. In both classes, the deterioration was clinically significant, i.e. in the higher class 6.1 and in the lower class 7.4 scores. The pace of deterioration was faster in the lower class leading to widening class inequalities around age 60 and plateauing after that. This widening was from 0.9 scores at the youngest ages to 2.2 scores at age 60.

Next, the analysis fitted three-variable interaction terms between age, class and employment status. The PCS trajectories by class for continuous employment, mandatory retirement and disability retirement are obtained from the same three-variable interaction model, but presented stratified in Figs. 2, 3 and 4 for clarity and comparison. Firstly, for those remaining employed (Fig. 2), the deterioration in functioning for the lower class was slightly faster than that in the full cohort (Fig. 1), and this deterioration continued until the highest ages without plateauing. The widening was from 1.2 at age 40 to 3.6 scores at age 67. After transition into mandatory retirement after the age of 55, the trajectories also declined for both classes (Fig. 3). The class inequalities were slightly narrower than for the employed, i.e. about 2.3 scores and statistically significant after age 60. The pace of deterioration in functioning was somewhat faster than for the employed, i.e. in the higher class from score 48.8 (46.6–51.1) at age 55 to 45.3 (44.2–46.3) at age 72, and in the lower class from 47.0 (44.2–49.9) at age 55 to 43.1 (41.9–44.2) at age 72. In both classes, the deterioration was clinically significant, i.e. in the higher class 3.5 and in the lower class 3.9 scores. Thirdly, after transition into disability retirement after the age of 50, the PCS score improved in both classes (Fig. 4), and contrasted with the pattern of deterioration among the employed and mandatorily retired. Starting from very poor levels (scores 34.6–35.3) among the youngest disability-retired, the PCS score improved strongly among those in older ages (scores up to 47). The improvement was highly clinically significant, i.e. over 10 scores. The higher class reported higher scores than the lower class until the oldest ages, when the class differences narrowed and no longer reached statistical significance.

Finally, considering simultaneously all trajectories in Figs. 2, 3 and 4, the deterioration of functioning for the continuously employed and the mandatorily retired, and the improvement for the disability retired led to convergence of functioning by age 65 for women in the different employment status transition groups, although with class inequalities remaining. As a result of the convergence, PCS scores for all three employment statuses were within 43–46.

## Discussion

We studied trajectories in functioning among Finnish women from midlife to retirement age and beyond. Our initial analyses focused on social class trajectories in physical functioning, as the participants become older. Our extended analyses examined whether employment status differentiation, i.e. continuous paid employment, transition to mandatory retirement or disability retirement would modify the class trajectories in functioning.

The main results can be summarised as follows. Firstly, among all women, physical functioning was better in the higher class than in the lower class and deteriorated faster in the lower class, leading to widening class trajectories by age 60 and plateauing after that. However, these overall trajectories obscured the divergent trajectories observed for the three employment statuses. Secondly, for continuously employed women, poorer functioning in the lower class deteriorated faster towards older age, leading to constant widening of the class trajectories. Thirdly, after transition to mandatory retirement at age 55 or older, functioning was also poorer in the lower class but deteriorated equally in both classes. Fourthly, after transition to disability retirement, the trajectories contrasted with the previous ones as functioning improved from initially poor level equally in both classes. Fifthly, simultaneous consideration of all three employment statuses showed convergent trajectories, with the disability-retired catching up the level of functioning among the employed and the mandatorily retired. Nevertheless, for each employment status group, the higher-class advantage in functioning either remained or strengthened.

The overall pattern of these results suggest the cumulative disadvantage model, with accumulating work exposures potentially contributing to ill health [21]. This finding has novel implications for understanding whether work is favourable or unfavourable towards to the end of work career [35]. In our study, exit from paid employment modified the class trajectories in functioning, allowing those who retire due to disability to catch up with those who follow the more favourable trajectories of higher-class employees remaining in work.

### Interpretation

Health and functioning typically deteriorate over adult life [20, 36, 37]. In our study, that was the case for the continuously employed and the mandatorily retired women among whom functioning deteriorated clinically significantly in both classes. However, after transition to disability retirement the initially poor functioning improved and reached the level of the continuously employed and the mandatorily retired by age 65, a common mandatory retirement age. The improvement was substantial and highly clinically significant.

Our follow up was 12 years, with ages ranging from 40 at Phase 1 to 72 years at Phase 3. Some prior studies suggest improvement in health after transition to retirement in general or to mandatory retirement [21, 22, 38, 39], whereas some others suggest deterioration towards retirement and beyond [13, 16, 20, 22, 37, 38]. This mixed picture calls for further analyses to add our understanding of the health trajectories and their potential explanations. Considering the bearing of social class brings the two theoretical models into the analysis, i.e. age-as-leveller suggesting narrowing or stable and cumulative disadvantage suggesting widening class trajectories in health [9–11].

Following, firstly, the age-as-leveller model, relative equalisation of disadvantage, such as work and non-work exposures, as well as advancing biological frailty in older ages would lead to narrowing class trajectories in functioning [11]. Our cohort can be assumed to be relatively advantaged, as all participants were employed at the beginning of the follow-up and they shared benefits like occupational health care and high employment security, which might speak for narrower class inequalities. Nevertheless, the class trajectories in functioning widened for the employed and remained after transition to both types of retirement, with some narrowing after disability retirement. A number of prior studies have equally reported widening or stable class trajectories in health in late working age and early old age [13, 15, 16, 39]. The evidence for the age-as-leveller effect is limited and might be seen only towards later old age [14, 17], not covered by our study.

Following, secondly, the cumulative disadvantage model, the class inequalities in the trajectories in functioning would widen, as disadvantage accumulates in an unequal way [9, 10]. In our study, widening materialised clearly for women remaining employed. This may be related, in particular, to accumulating work exposures [21]. There were minor signs for widening also after mandatorily retirement, but no signs after disability retirement. Mandatory retirement follows a “normal” route to exit paid employment, with no necessary disadvantage, and this may lie behind the almost equal deterioration of functioning in both classes. The route to retirement due to work disability, in turn, is a lengthy process starting years before statutory retirement age. Work disability results from mismatch between individual characteristics, i.e. loss of health and functioning, in particular, as well as accumulating work exposures [30, 40]. This double nature of work (dis)ability may help understand the poor initial functioning among the disability-retired, its subsequent improvement as well as the stability of the class inequalities in the trajectories in our study. The improvement in functioning found after transition to disability retirement may be an indication of the benefits of the prior rehabilitation and treatment efforts as well as other benefits of being eligible for disability retirement equally in the lower and the higher class, as suggested also by a Swedish study [4].

Studies show some evidence for the cumulative disadvantage model but less for the age-as-leveller model [13, 14, 16, 19]. A reason for the unclear status of the theoretical models vis-à-vis evidence may relate to their general nature. The models predict overall socioeconomic inequalities in health trajectories [9–11], whereas subgroups behind and divergent routes to exit paid employment are not captured. Our analyses, based on three-way interaction between age, social class and employment status, suggest divergent trajectories for the subgroups. Transition to retirement, in particular, and social class, to a lesser extent, modified the trajectories in functioning among originally employed women from midlife to post-retirement age. Yet, the explanatory power of the two theoretical models may be better visible in later old age.

Examining the joint importance of age, social class and employment status for functioning, a major novel finding of our study was that retirement type, i.e. transition to either mandatory or disability retirement, modified the class trajectories in functioning during ageing. The most striking modifying effect was the contrast between the deterioration of functioning after mandatory retirement but improvement after disability retirement. There is some prior evidence from Britain on retirement types among non-manual classes that is in accordance with our findings [20]. Finally, considering simultaneously all trajectories, functioning converged between the continuously employed, the mandatorily retired and the disability-retired women, and reached an equal level of functioning by age 65. The prior evidence is also in accordance with the convergent trajectories that we found [20]. Throughout, the class inequalities in functioning among the studied women remained for the three employment statuses towards retirement age, suggesting that the inequalities in functioning are deep-rooted, like health inequalities in general [41].

### Methodological considerations

Our data came from a relatively large prospective cohort, collected at Phases 1–3 among originally employed women. The study design was suitable for our purposes to examine class trajectories in functioning among employees facing transition to retirement. We measured SF-36 physical functioning, which is a reliable and validated instrument [31].

There were limitations as well. Firstly, non-response and attrition are inherent problems in follow-up surveys [42]. Response to our surveys was acceptable or good. Our non-response analyses have shown that the data are largely representative of the target population, with lower class responders somewhat underrepresented at baseline [25]. Further analyses including Phases 1–3 showed that the class difference in responding was minimal for ages below 60 and somewhat larger for ages above 60. Class mobility over Phases 1–3 was minor and more often upwards than downwards; overall, 90% remained in the same class. While lower class responders had poorer functioning than their higher-class counterparts, this difference was similar between the responders and the non-responders. Employment status distributions over age were largely similar in both classes, but after age 55, the proportion of disability-retired was higher in the lower class whereas the proportion of mandatorily retired was higher in the higher class. The effects of non-response and attrition are likely modest and conservative [42]. Nevertheless, we acknowledge these are potential sources of bias. Secondly, 80% of our respondents were women, which corresponds to the Finnish municipal sector. The female majority in our data allowed us to focus on women only, whereas many prior studies have been male-dominated or included exclusively men. Sensitivity analyses among men suggested somewhat less steep deterioration and narrower class inequalities in functioning compared to women. However, the number of men was too small for reliable analyses of the modifying effect of employment status. Thirdly, we adjusted for working hours as well as period and cohort in sensitivity analyses, but these had negligible effects on the trajectories. Fourthly, further sensitivity analyses used four social classes instead of two, but the results were practically identical. Only for employed women, four classes showed slightly stronger widening of the class trajectories in functioning. Fifthly, selective mortality may constrain the widening of the class trajectories, as those in lower classes have poorer health and higher mortality [16]. Maximal age among our participants was 72 years and this limits the number of deaths. Sixthly, our retirement variable derived from survey questionnaire, which lacked details on part-time retirement and diagnoses for disability retirement. Finally, our results cannot be directly generalised to other countries, the Finnish population or labour force at large, but they represent better public sector employees and, in particular, municipal employees.

## Conclusions

We found persisting social class inequalities in the trajectories of physical functioning among ageing Finnish women originally employed, but employment status, i.e. remaining employed, transitioning to mandatory or disability retirement, modified these trajectories. Functioning deteriorated with ageing among those who remained in paid employment or retired mandatorily, with some evidence of widening class inequalities. In contrast, after transition to disability retirement, initial poor functioning improved and led to convergence of functioning between the three employment statuses towards older ages. The overall pattern of the results suggest evidence for the cumulative disadvantage model, with accumulating work exposures among disadvantaged classes potentially contributing to their trajectories of ill health.

## Data Availability

The datasets have been generated by the Helsinki Health Study, University of Helsinki, and are kept at the University of Helsinki IT Center in protected computers. The datasets contain register data that are open only on permission from the register keepers. The longitudinal survey data are available for research purposes on reasonable request from the Helsinki Health Study.

## Abbreviations

PCS: Physical component summary
SF-36: Short Form 36
